# New taxa of quill mites (Acariformes: Syringophilidae) parasitising aquatic birds of the Faroe Islands

**DOI:** 10.1007/s11230-024-10182-z

**Published:** 2024-08-20

**Authors:** Maciej Skoracki, Simon Haarder

**Affiliations:** 1 Department of Animal Morphology, Faculty of Biology, Adam Mickiewicz University, 61-614 Poznań, Poland; 2 Ornebjergvej 43, 4760 Vordingborg, Denmark

## Abstract

The paper presents descriptions of new taxa and new records of quill mites of the family Syringophilidae Lavoipierre, 1953 (Acari: Prostigmata: Cheyletoidea) parasitising aquatic birds in the Faroe Islands, Denmark. *Sulisyringophilus jenskjeldi* **n. gen.**, **n. sp.**, is described from the northern gannet, *Morus bassanus* (Linnaeus) (Suliformes: Sulidae). The new genus, *Sulisyringophilus*, is new genus differs from the morphologically similar genus *Procellariisyringophilus* Schmidt and Skoracki, 2007 by the presence of lateral hypostomal teeth and leg setae *vsII* in females, the features which are absent in the latter. A new species *Charadriphilus lymnocryptes* **n. sp.** is described from the jack snipe, *Lymnocryptes minimus* (Brünnich) (Charadriiformes: Scolopacidae). Additionally, two rarely recorded species, *Niglarobia ereuneti* Kethley, 1970, and *Creagonycha lara* Kethley, 1970, are reported from two charadriiform hosts: the semipalmated sandpiper, *Calidris pusilla* (Linnaeus) and the black-legged kittiwake, *Rissa tridactyla* (Linnaeus), respectively.

## Introduction

Research on parasites associated with vertebrate hosts on islands and archipelagos is of particular interest, as it plays a crucial role in understanding parasitic behavior and ecosystem development in relatively isolated conditions.Among the specialised parasites associated with birds, the quill mites of the family Syringophilidae (Acariformes: Prostigmata: Cheyletoidea) are distinguished by their high morphological specialisation. These mites inhabit exclusively the internal cavities of feather quills, carrying out their entire lifecycle within this unique microhabitat, including feeding and reproduction. The family Syringophilidae represents the most taxonomically diverse group among quill-inhabiting mites, occupying a wide range of habitats in the plumage of their hosts (Kethley, 1970; Skoracki, 2011). Despite their diversity, the taxonomic system of syringophilid mites remains unsatisfactorily developed. Currently, the family comprises approximately 400 described species, and its representatives have been recorded from hosts representing 27 bird orders (Zmudzinski et al., 2023).

The Faroe Islands, an archipelago in the North Atlantic Ocean, consist of 18 volcanic islands. Their unique geological features and geographical location make them a significant habitat for a wide array of seabirds and waders, which either breed on or regularly visit the islands. A comprehensive survey has documented a total of 355 bird species on the Faroe Islands, including subspecies and introduced species (Olofson & Sørensen, 2022). Of these, approximately 150 species are aquatic birds belonging to the orders Anseriformes, Charadriiformes, Gruiformes, Procellariiformes, and Suliformes (Jensen & Sørensen, 2015). Notably, Syringophilidae mites have never been studied in this region before, marking this investigation as particularly pioneering. Consequently, this paper introduces the initial study on syringophilid mites of birds from the Faroe Islands, with a special focus on aquatic birds. The syringophilid mites parasitising this host group comprise approximately 30 species grouped in 16 genera of the subfamily Syringophilinae and three species in two genera of the subfamily Picobiinae (Kethley, 1970; Skoracki, 2011; Glowska & Skoracki, 2011; Glowska, 2015; Skoracki & Zawierucha, 2016; Skoracki et al., 2006, 2016, 2017, 2022; Zmudzinski & Skoracki, 2017; Zmudzinski et al., 2016, 2018). These mite genera have been recorded on birds representing seven orders: Anseriformes, Charadriiformes, Gruiformes, Phoenicopteriformes, Procellariiformes, Suliformes, and Pelecaniformes.

**Fig. 1:**
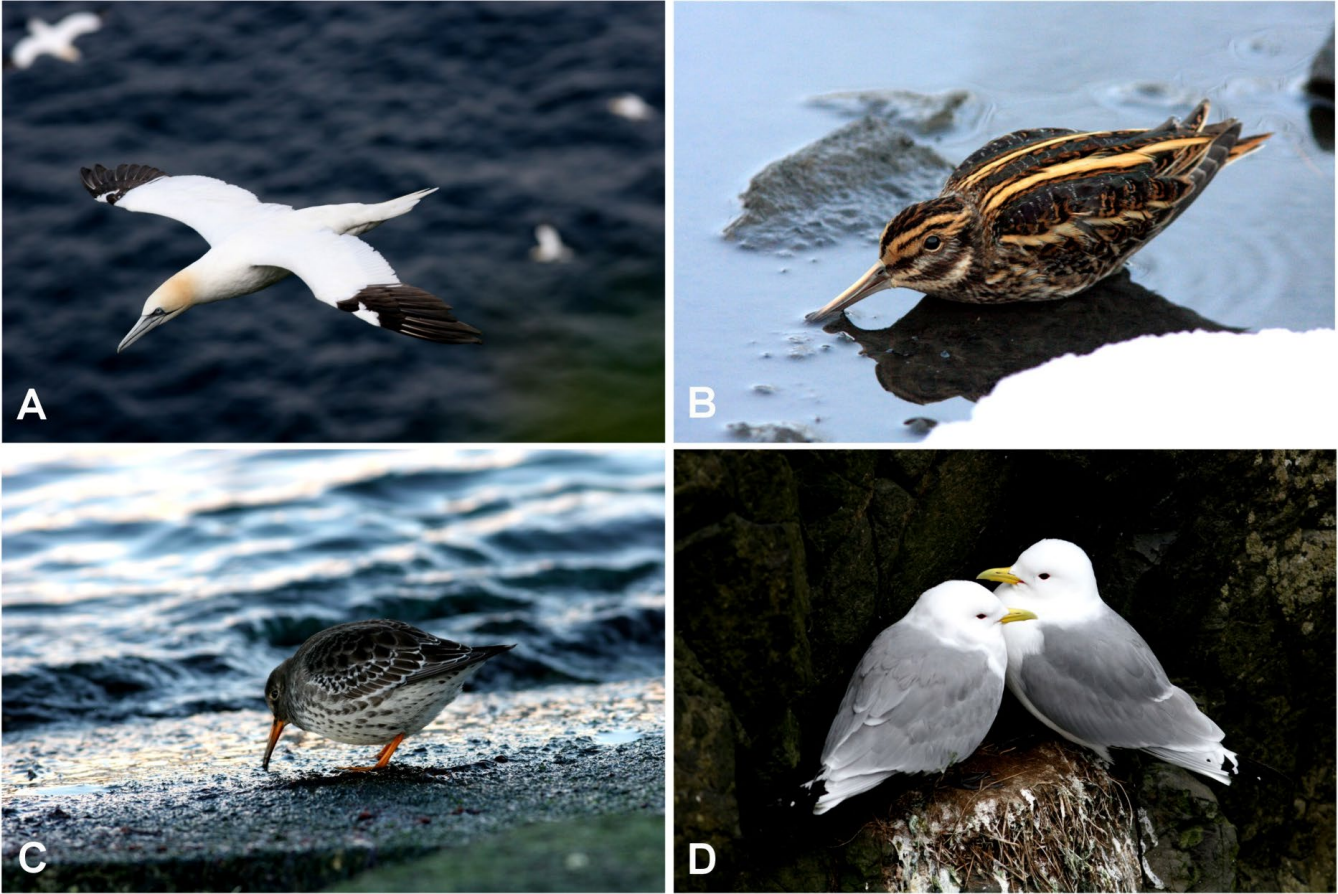
Host species infested by quill mites: **A**, *Morus bassanus*; **B**, *Lymnocryptes minimus*; **C**, *Calidris pusilla*; **D**, *Rissa tridactyla*. Photos by Jens-Kjeld Jensen (**A**, **C**, **D**), and Ingvar A. Sigurðsson (**B**).

**Fig. 2:**
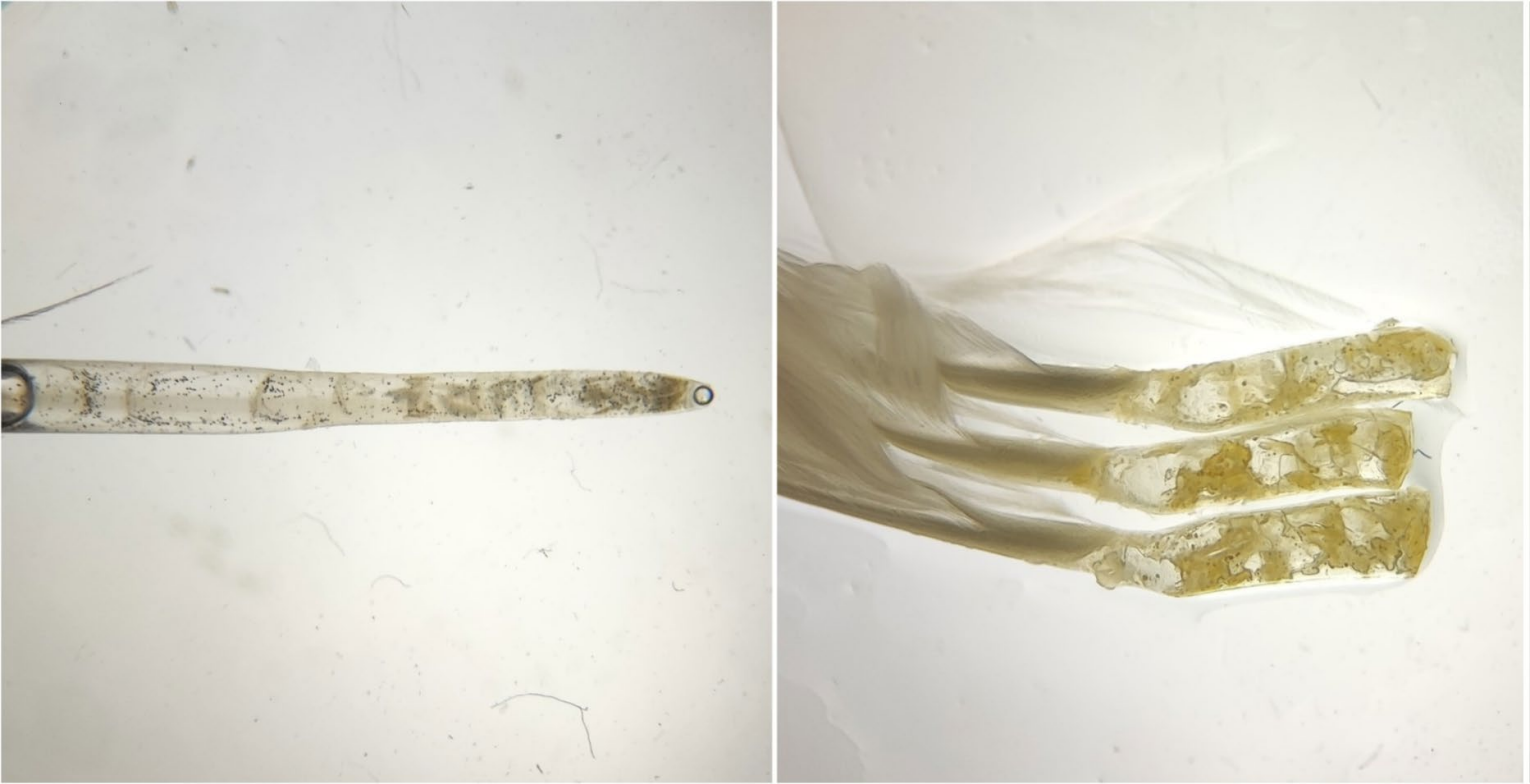
Examples of infested feather quills of *Lymnocryptes minimus* (left), *Morus bassanus* (right). Photo by Simon Haarder.

In this paper, we present results of our study of syringophilid mites, based on 75 specimens of 31 species of aquatic birds from the Faroe Islands. We establish a new genus, *Sulisyringophilus* **n. g.**, based on a newly described species, *Sulisyringophilus jenskjeldi* **n. sp.**, from the northern gannet, *Morus bassanus* (Linnaeus) (Suliformes: Sulidae). Furthermore, we describe a new species*Charadriiphilus lymnocryptes* **n. sp.**, from the jack snipe, *Lymnocryptes minimus* (Brünnich) (Charadriiformes: Scolopacidae). In this study, we also added the new host records for two previously described quill mite species from charadriiform hosts: the semipalmated sandpiper *Calidris pusilla* (Linnaeus) (Scolopacidae) for *Niglarobia ereuneti* Kethley, 1970 and the black-legged kittiwake *Rissa tridactyla* (Linnaeus) (Laridae) for *Creagonycha lara* Kethley, 1970 (Figs. 1, 2).

## Materials and methods

The birds were collected by Jens-Kjeld Jensen (Nólsoy, Faroe Islands, Denmark) over the period from 1996 to 2024 and were preserved in his private freezer until examination. Their causes of death varied, including natural causes, traffic accidents, predation by feral cats, or hunting. For each bird studied, the majority of the flight, tail, and covert feathers were meticulously removed and examined for the presence of quill mites. Additionally, 10–20 contour feathers from the chest and ventral areas were carefully extracted and analysed. An overview of examined bird species is presented in Table 1.

**Table 1:** Examined bird species from the Faroe Islands and their quill mites

Bird species	Order and family	No. examined birds	No. infested birds	Quill mite species
*Anser anser*	Anseriformes: Anatidae	1	–	
*Clangula hyemalis*	Anseriformes: Anatidae	1	–	
*Cygnus cygnus*	Anseriformes: Anatidae	2	–	
*Somateria mollissima faeroeensis*	Anseriformes: Anatidae		2 –	
*Alca torda*	Charadriiformes: Alcidae	1	–	
*Alle alle*	Charadriiformes: Alcidae	3	–	
*Cepphus grylle faeroeensis*	Charadriiformes: Alcidae	2	–	
*Fratercula arctica*	Charadriiformes: Alcidae	5	–	
*Uria aalge*	Charadriiformes: Alcidae	1	–	
*Uria lomvia*	Charadriiformes: Alcidae	1	–	
*Haematopus ostralegus*	Charadriiformes: Haematopodidae	3	–	
*Larus canus*	Charadriiformes: Laridae	1	–	
*Larus fuscus*	Charadriiformes: Laridae	1	–	
*Larus glaucoides*	Charadriiformes: Laridae	2	–	
*Rissa tridactyla*	Charadriiformes: Laridae	20	1	*Creagonycha lara*
*Sterna paradisaea*	Charadriiformes: Laridae	2	–	
*Calidris alpina schinzii*	Charadriiformes: Scolopacidae	1	–	
*Calidris maritima*	Charadriiformes: Scolopacidae	2	2	*Niglarobia ereuneti*
*Gallinago gallinago*	Charadriiformes: Scolopacidae	1	–	
*Lymnocryptes minimus*	Charadriiformes: Scolopacidae	3	1	*Charadriphilus lymnocryptes*
*Stercorarius parasiticus*	Charadriiformes: Stercorariidae	1	–	
*Stercorarius skua*	Charadriiformes: Stercorariidae	2	–	
*Gallinula chloropus*	Gruiformes: Rallidae	1	–	
*Rallus aquaticus*	Gruiformes: Rallidae	1	–	
*Hydrobates pelagicus*	Procellariiformes: Hydrobatidae	2	–	
*Fulmarus glacialis*	Procellariiformes: Procellariidae	4	–	
*Puffinus* (*Ardenna*)* gravis*	Procellariiformes: Procellariidae	2	–	
*Puffinus* (*Ardenna*)* griseus*	Procellariiformes: Procellariidae	3	–	
*Puffinus puffinus*	Procellariiformes: Procellariidae	1	–	
*Phalacrocorax aristotelis*	Suliformes: Phalacrocoracidae	2	–	
*Morus bassanus*	Suliformes: Sulidae	1	1	*Sulisyringophilus jenskjeldi*
Total		75	5	

Mites found in infested feathers were meticulously removed using fine-pointed tweezers. These specimens underwent a clearing and softening process by being immersed in Nesbitt’s solution at ambient temperature for 24 to 36 hours (Skoracki, 2011). Subsequently, mites were transferred to 70% ethanol for a brief period of approximately 10 minutes and then mounted on microscopic slides using Hoyer’s medium, following the established protocol by Krantz and Walter (2009). The mite specimens were examined using a ZEISS Axioscope light microscope equipped with differential interference contrast (DIC) optics. Illustrations were made using a camera lucida attachment. All measurements are given in micrometres, with the dimension ranges of paratypes presented in parentheses, following the measurements of the holotype. The idiosomal setation nomenclature aligns with Grandjean’s (1939) system as modified for Prostigmata by Kethley (1990), and the leg chaetotaxy follows Grandjean’s (1944) classification. All other morphological terminology follows Kethley (1970) and Skoracki (2011).

Specimen depositories are cited using the following abbreviations: AMU, A. Mickiewicz University, Department of Animal Morphology, Poznan, Poland; NHMD, Natural History Museum of Denmark, Copenhagen, Denmark.

**Family** ***Syringophilidae*** **Lavoipierre, 1953**

**Subfamily Syringophilinae Lavoipierre, 1953**

***Sulisyringophilus*** **n. g.**

Diagnosis

Female. Large-sized syringophilids (total body length 960–1090). *Gnathosoma*. Lateral hypostomal teeth present. Peritremes M-shaped with clearly visible chambers. Movable cheliceral digit edentate. Stylophore constricted posteriorly, ended by projection. *Idiosoma*. Six pairs of discernible ornamented propodonotal setae arranged 3–1–2. Propodonotal shield entire, without pocket-like structures, bearing bases of setae *vi*, *ve*, *si*, *c1* and *se*. Hysteronotal shield absent. Hysteronotal setae *d1*, *d2* and *e2* long. Setae *d1* closer to *d2* than to *e2*. Pygidial shield present. Terminal setae *f2* and *h2* long, *f1* and *h1* short. Pseudanal setal series with 2 pairs of setae (*ps1–2*). Genital setal series represented by 1 pair of setae (*g2* absent). Aggenital series with 3 pairs of setae (*ag1–3*). *Legs*. Legs I thicker than II–IV. Antaxial and paraxial members of claw pair subequal in size and shape. Coxal apodemes I slightly divergent, not fused to apodemes II. Legs with full complement of setae.

Male. Characteristics as in female except: total body length 660; lateral hypostomal teeth absent; posterior margin of stylophore without projection; all setae on propodonotum smooth; setal pattern of propodonotal region arranged 3–2–1; propodonotal shield bearing bases of setae *vi*, *ve*, *si* and *c1*; hysteronotal shield fused to pygidial shied; hysteronotal setae short; setae *f2* short, *h2* long; genital setal series represented by 2 pairs of setae; legs I–IV equal in thickness; coxal apodemes I strongly divergent.

*Type-species*: *Sulisyringophilus jenskjeldi* **n. sp.**

*ZooBank registration*: The Life Science Identifier (LSID) for *Sulisyringophilus* **n. g.** is urn:lsid:zoobank.org:act:E1D3498F-0E1A-4D97-9347-8635FB3524A9

*Etymology*: The name refers to the order name of the host - Suliformes, and *Syringophilus* - type genus of the family Syringophilidae.

### Remarks

This new genus is morphologically close to the monotypic genus *Procellariisyringophilus* Schmidt and Skoracki, 2007. In females of both genera, the movable chericeral digits are edentate; the peritremes are M-shaped; the stylophore is constricted posteriorly; idiosomal setae are stout and strongly ornamented; the hysteronotal shield is absent; the pygidial shield is present; the genital setal series is represented by 1 pair of setae; the pseudanal setal series with 2 pairs of setae, and the coxal apodemes I are weakly divergent. *Sulisyringophilus* differs from *Procellariisyringophilus* in the following features: in females of *Sulisyringophilus*, the lateral hypostomal teeth and leg setae *vsII* are present. In females of *Procellariisyringophilus*, the lateral hypostomal teeth and leg setae *vsII* are absent. Furthermore, the new genus can be easily distinguished from *Stibarokris* Kethley, 1970, which females also possess strongly ornamented idiosomal setae and lateral hypostomal teeth, by the presence of only 1 pair of genital setae (*vs.* 2 pairs in *Stibarokris*).

***Sulisyringophilus jenskjeldi*** **n. sp.**

*Type-host*: *Morus bassanus* (Linnaeus) (Suliformes: Sulidae), northern gannet.

*Type-locality*: Nólsoy, Faroe Islands.

*Type-material*: Female holotype, 11 female and 1 male paratypes (reg. no. MS-24-0124-001), 1 September 2021, mites collected by Simon Haarder, bird obtained by Jens-Kjeld Jensen.

*Site in host*: Quills of contour feathers.

*Type material deposition*: Female holotype and most paratypes will be deposited in the AMU, except four female paratypes in the NHMD.

*ZooBank registration*: The Life Science Identifier (LSID) for *Sulisyringophilus jenskjeldi* **n. g., sp.** is urn:lsid:zoobank.org:act:A0C755AC-39E3-460F-9AC7-34FB69FE57C6

*Etymology*: This species is named in the honour of Jens-Kjeld Jensen, the renowned Faroese ornithologist, naturalist and taxidermist.

### Description (Figs. 3, 4, 5, 6)

**Fig. 3:**
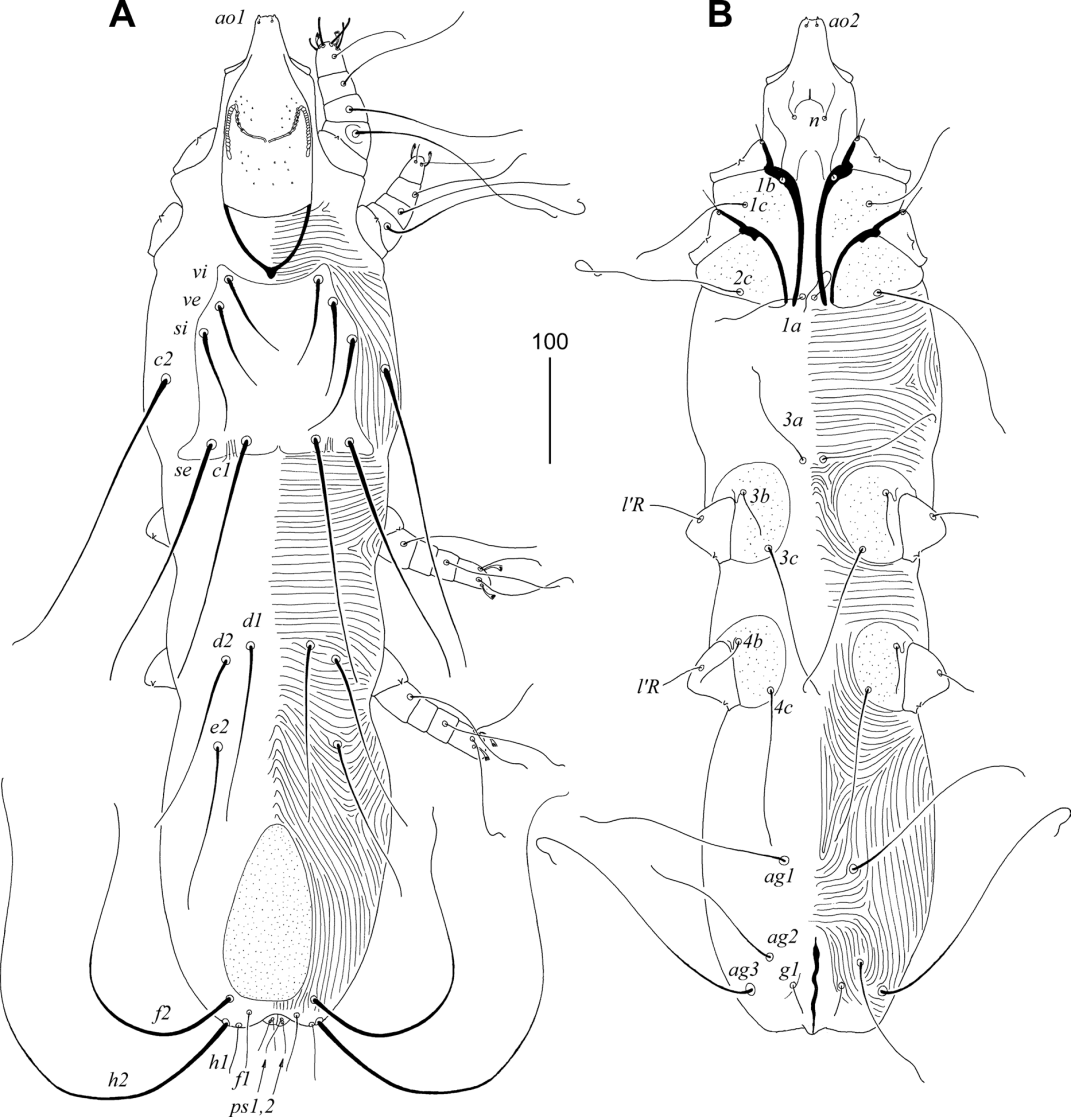
*Sulisyringophilus jenskjeldi* **n. sp.** Female. A, Dorsal view; B, Ventral view. *Scale-bar*: 100 µm.

**Fig. 4:**
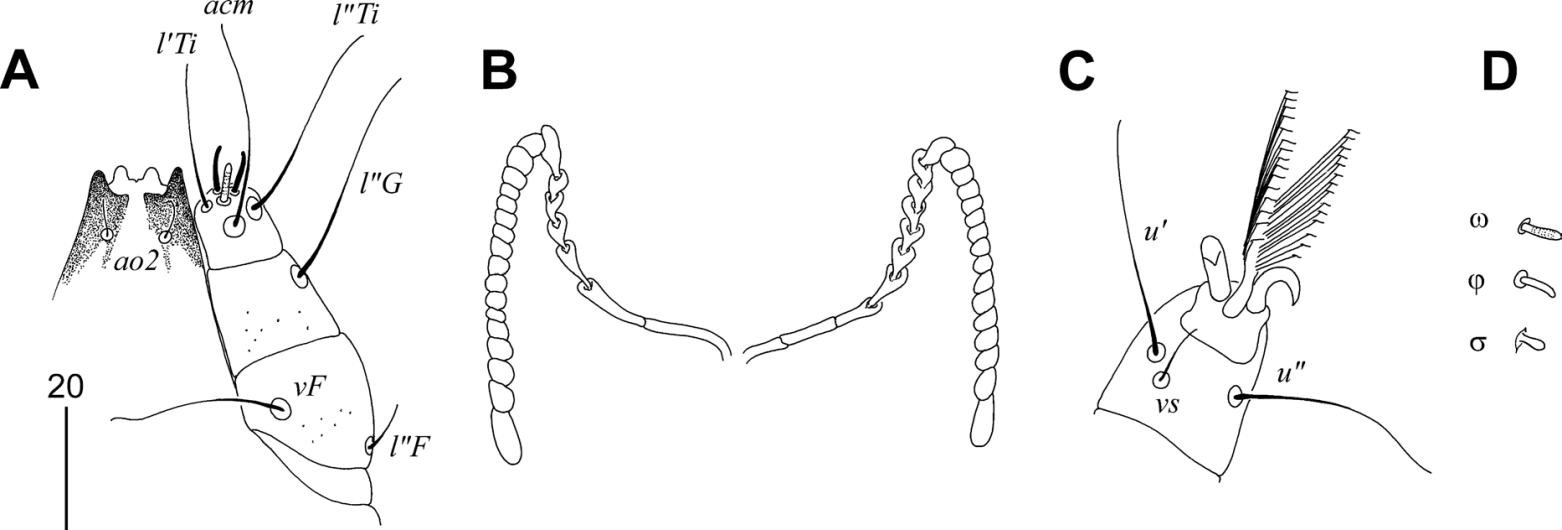
*Sulisyringophilus jenskjeldi* **n. sp.** Female. A, Gnathosoma in ventral view; B, Peritremes; C, Tarsus II; D, Solenidia of leg I. *Scale-bar*: 20 µm.

Female. Total body length 980 in holotype (range for 11 paratypes 960–1090). *Gnathosoma*. Hypostomal apex with pair of strongly sclerotised and blunt-ended hypostomal teeth (Fig. 4A). Each medial branch of peritremes with 8–9 chambers, each lateral branch with 16–17 chambers (Fig. 4B). Stylophore constricted posteriorly, 265 (255–265) long; exposed portion of stylophore apunctate, 200 (200–205) long; posterior margin of stylophore with projection. Movable cheliceral digit, 200 (200) long. *Idiosoma*. All dorsal setae thick and ornamented, opishonotal setae *f1*, *f2* short and smooth, *f2* and *h2* long, smooth and whip-like. Propodonotal shield well sclerotised, punctate in anterior half, and slightly concave on anterior margin. Propodonotal setae *vi*, *ve* and *si* subequal in length. Hysteronotal shield absent. Bases of setae *d1* situated slightly anterior to level of setal bases *d2*. Pygidial shield egg-shaped, well sclerotised, densely punctate; bases of setae *f1* and *f2* not situated on pygidial shield. Bases of setae *1a* and *2c* situated on same transverse level; bases *3a* situated anterior to level of setal bases *3b*. Coxal apodemes I slightly divergent, not fused to apodemes II. All coxal fields punctate. Genital plate absent. Both pairs of pseudanal setae (*ps1–2*) subequal in length. Genital setae short. Body cuticular striations as in Fig. 3. *Legs*. Femur, genu and trochanter of legs I punctate ventrally. Tarsal claws normally developed, both similar in shape and size (Fig. 4C). Fan-like setae *p'* and *p"* of trasi III–IV with reduced number of tines, 5–6. Solenidia *ωI* and *φI* equal in length, both slightly longer than *σI* (Fig. 4D). All tarsal claws without basal angle. Lengths of setae: *vi* 90 (70–90), *ve* 100 (80–85), *si* 90 (80–100), *se* (230–260), *c1* (230), *c2* 295 (250–285), *d1* 200 (170–190), *d2* (170–180), *e2* 140 (140–175), *f1* 50 (50), *f2* 335 (340–370), *h1* 50 (50), *h2* 490 (485–490), *g1* 25 (30–40), *ps1* and *ps2* 30 (30–35), *ag1* 220 (180–200), *ag2* 135 (145–155), *ag3* 300 (260–275), *1a* (165), *3a* (110), *3b* 50 (50), *4b* 55 (45–70), *3c* (115–140), *4c* 145 (105–145), *l'RI* (8), *l'RII* (10), *l'RIII* 55 (45–50), *l'RIV* 35 (30–40), *tc'III–IV* 80 (80–95), *tc"III–IV* 105 (95–115).

**Fig. 5:**
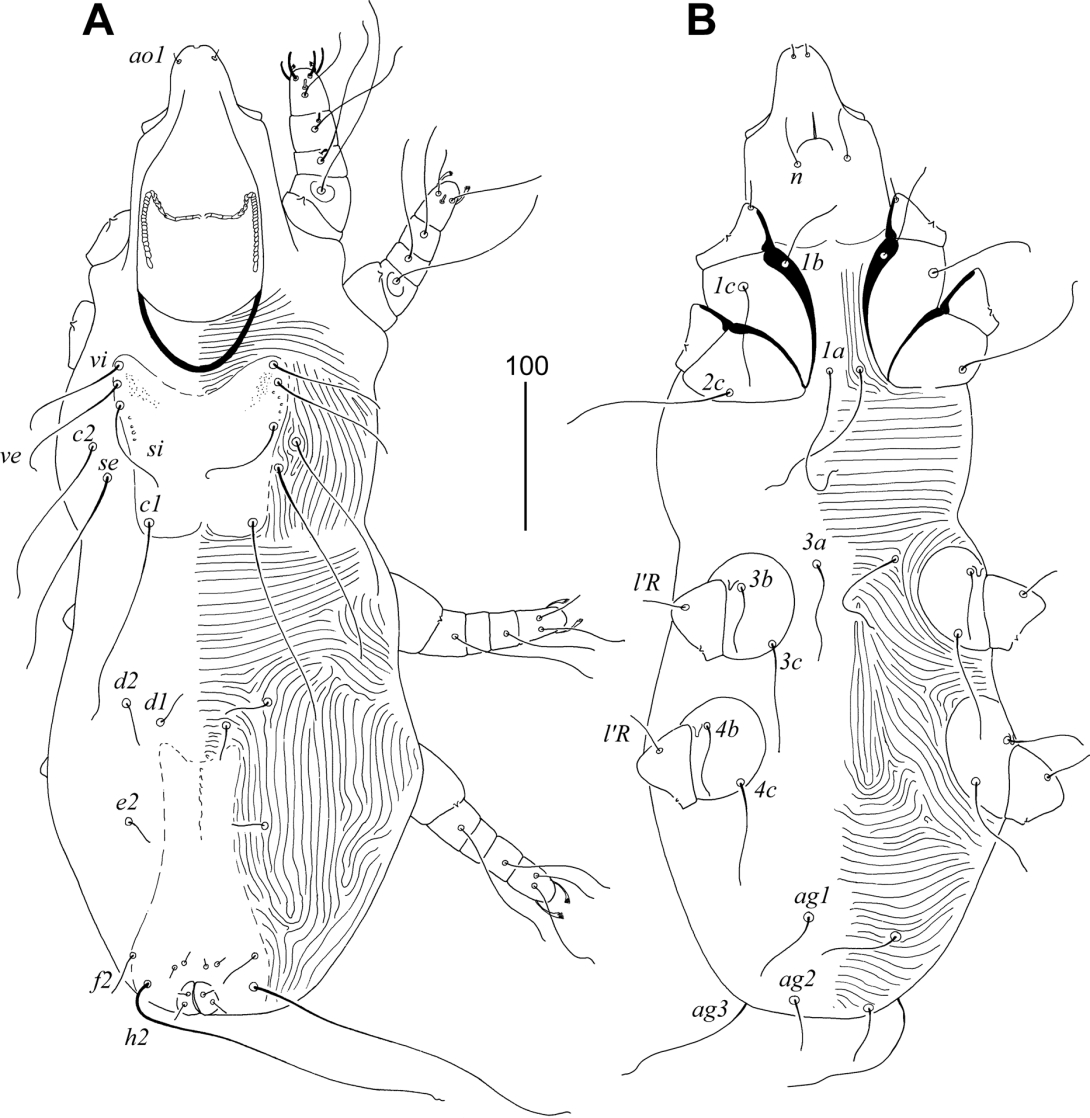
*Sulisyringophilus jenskjeldi* **n. sp.** Male. A, Dorsal view; B, Ventral view. *Scale-bar*: 100 µm.

**Fig. 6:**
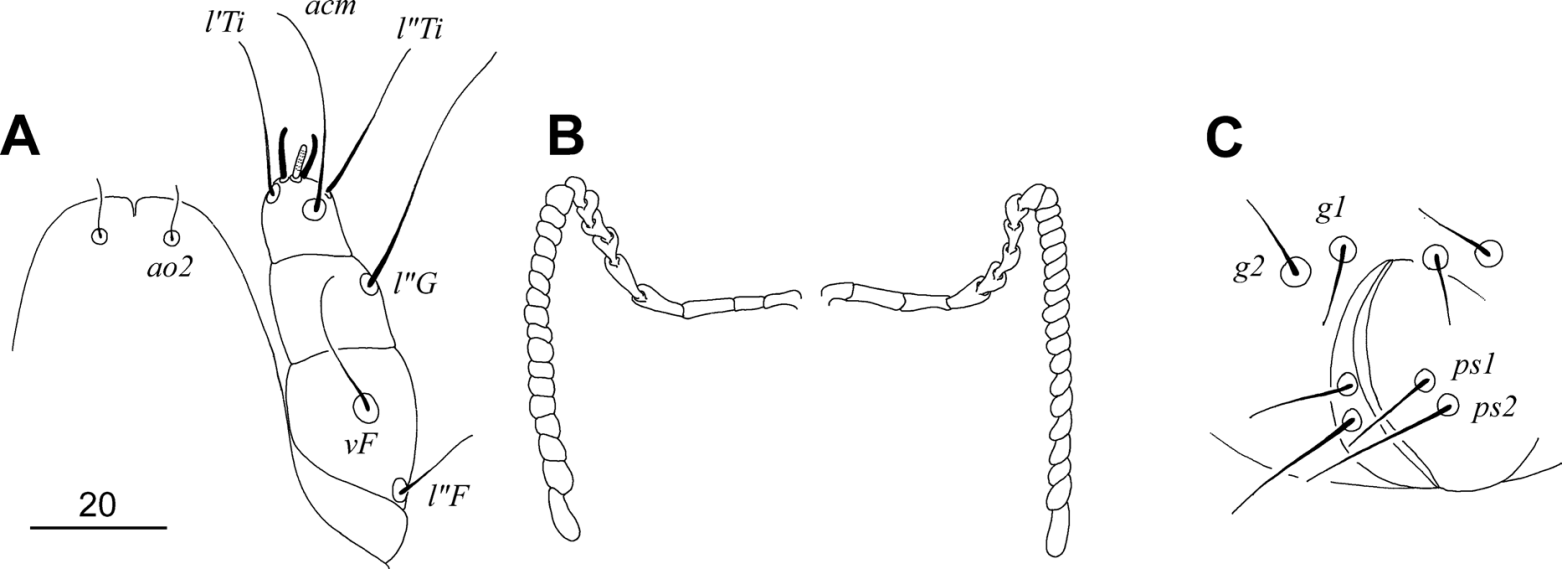
*Sulisyringophilus jenskjeldi* **n. sp.** Male. A, Gnathosoma in ventral view; B, Peritremes; C, genito-anal opening. *Scale-bar*: 20 µm.

Male. Total body length 660 (in 1 paratype). *Gnathosoma*. Hypostomal apex rounded, without protuberances (Fig. 6A). Infracapitulum apunctate, 140 long. Stylophore rounded posteriorly, 220 long; exposed portion of stylophore apunctate, 190 long. Each medial branch of peritremes with 8–9 chambers, each lateral branch with 17–20 chambers (Fig. 6B). *Idiosoma*. Propodonotal shield well sclerotised, concave on anterior margin, punctate, bearing bases of setae *vi*, *ve*, *si* and *c1*. Bases of setae *c2* situated posterior to level of setal bases *si* and anterior to bases of setae *se*. Setal bases *se* situated anterior to *c1*. Hysteronotal shield apunctate, fused with pygidial shield, reaching anteriorly above level of setal bases *e2*, bearing only bases of setae *f2* and *h2*. Bases of setae *d1* situated closer to *d2* than to *e2*. Bases of setae *1a* situated slightly anterior to level of setal bases *2c*, bases *3a* situated slightly anterior to level of setal bases *3b*. Coxal fields I–IV well sclerotised, apunctate. Three pairs of aggenital setae present. Setae *g1* and *g2* subequal in length and situated on same transverse level (Fig. 6C). Setae *ps2* longer than *ps1*. Cuticular striations as in Fig. 5. *Legs*. Both tarsal claws of legs I–IV of same size and shape. Lengths of setae: *vi* 90, *ve* 100, *si* 60, *se* 140, *c1* 135, *c2* longer than 100, *d1* 20, *d2* 30, *e2* 20, *f2* 20, *h2* 230, *ag1* 65, *ag2* 35, *ag3* 85.

***Charadriphilus lymnocryptes*** **n. sp.**

*Type-host*: *Lymnocryptes minimus* (Brünnich) (Charadriiformes: Scolopacidae), jack snipe.

*Type-locality*: Tórshavn, Faroe Islands.

*Type-material*: Female holotype and 13 female and 5 male paratypes (reg. no. MS-23-0808-001), 13 September 2019, mites collected by Simon Haarder, bird obtained by Jens-Kjeld Jensen.

*Site in host*: Quills of secondaries.

*Type material deposition*: Holotype and most paratypes will be deposited in the AMU, except 5 female and 2 male paratypes in the NHMD.

*ZooBank registration*: The Life Science Identifier (LSID) for *Charadriphilus lymnocryptes* **n. sp.** is urn:lsid:zoobank.org:act:8DC86654-42F8-4631-A64D-17EC50FF4A5E

*Etymology*: The species name “*lymnocryptes*” is taken from the generic name of the host.

### Description (Figs. 7, 8)

**Fig. 7:**
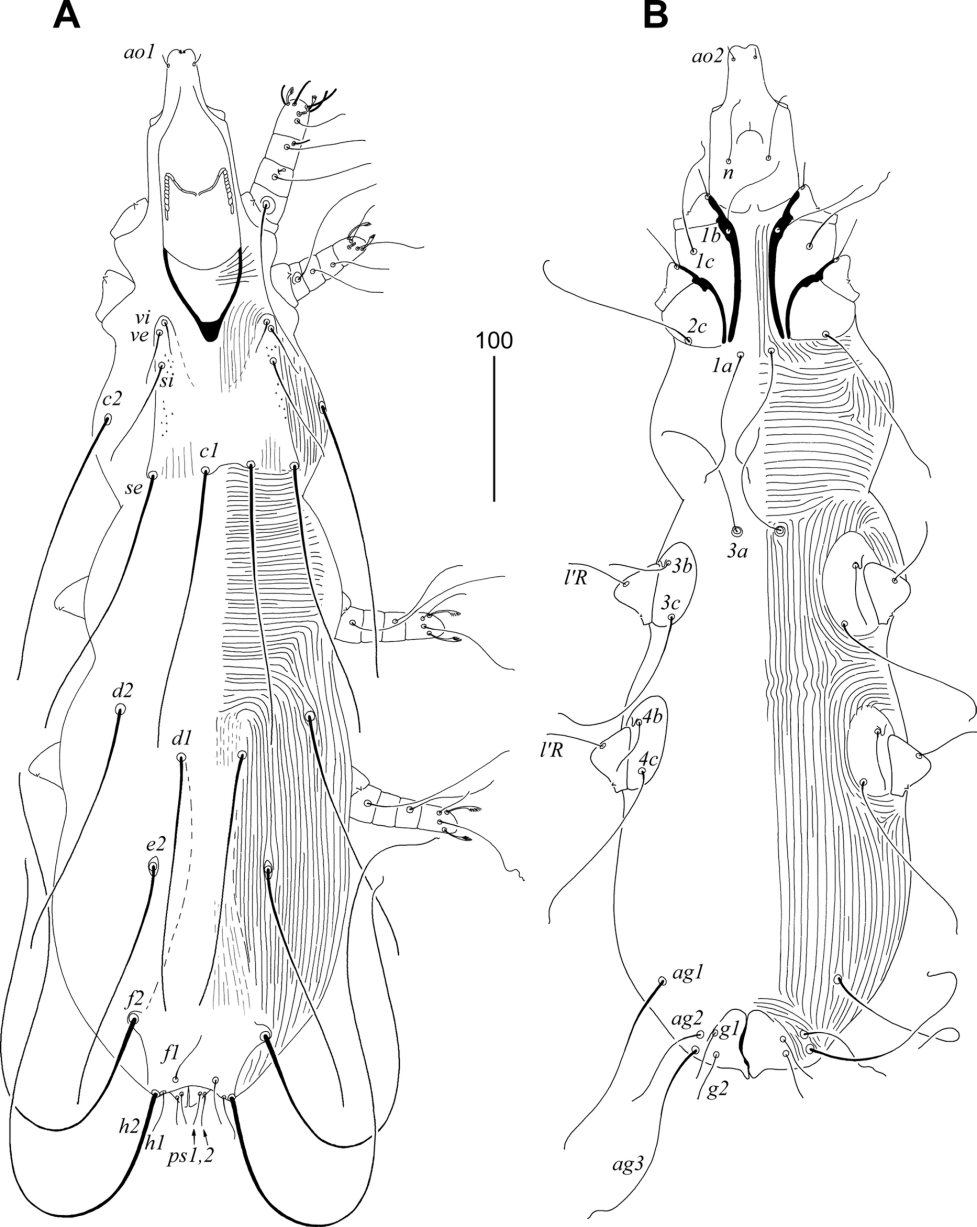
*Charadriphilus lymnocryptes* **n. sp.** Female. A, Dorsal view; B, Ventral view. *Scale-bar*: 100 µm.

**Fig. 8:**
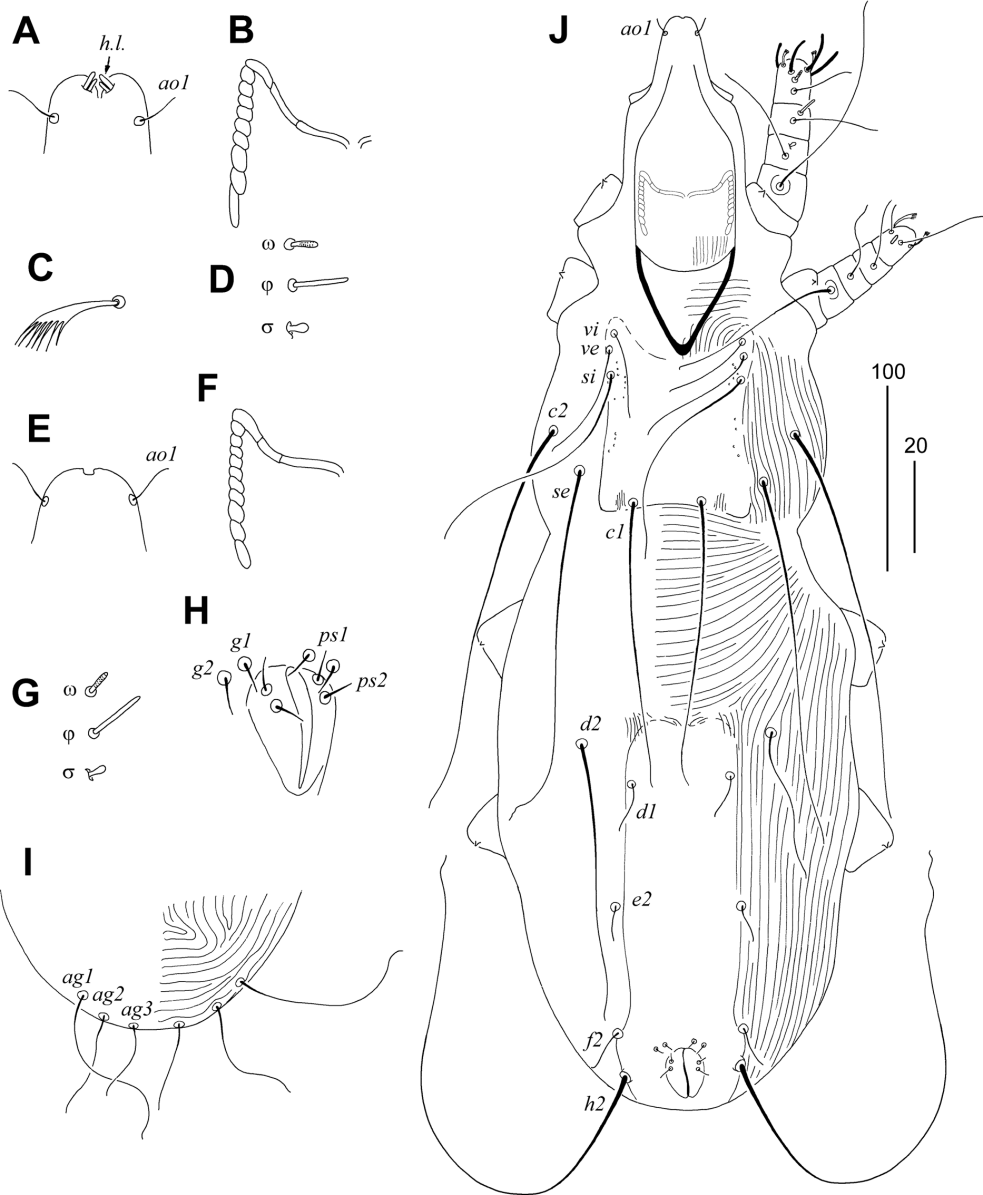
*Charadriphilus lymnocryptes* **n. sp.** Female (A–D). A, Hypostomal apex in dorsal view; B, Peritreme; C, fan-like seta *p'III*; D, solenidia of leg I. Male (E–J). E, Hypostomal apex in dorsal view; F, Peritreme; G, solenidia of leg I; H, genito-anal opening; I, opisthosoma in ventral view; J, Body in dorsal view. *Scale-bars*: 20 µm (A–H), 100 µm (I, J).

Female. Total body length 725 in holotype (range for 13 paratypes 700–730). *Gnathosoma*. Infracapitulum apunctate. Hypostomal apex rounded and smooth; 2 pairs of large hypostomal lips present (Fig. 8A). Movable cheliceral digits 160 (160–165) long. Stylophore constricted posteriorly, 200 (200–220) long; exposed portion of stylophore apunctate, 155 (155–165) long. Each medial branch of peritremes with 3 chambers, each lateral branch with 9 chambers (Fig. 8B). *Idiosoma*. Propodonotal shield punctate laterally, weakly sclerotised, concave on anterior margin, bearing bases of all propodonotal setae except *c2*. Bases of setae *c1* and *se* situated on same transverse level. Length ratio of setae *vi*:*ve*:*si* 1:1.4–1.5:2.8–3. Hysteronotal shield fused with pygidial shield, weakly sclerotised in anterior part and between bases of setae *e2* and *f2*, apunctate, bearing bases of setae *d1*, *f1* and *f2*. All coxal fields apunctate. Length ratio of setae *ag1*:*ag2*:*ag3* 1.3–1.7:1:1.7–1.9. Both pairs of genital setae subequal in length and situated on genital plate. Cuticular striations as in Fig. 7. Legs. Fan-like setae *p'* and *p"* of legs III–IV with 6–8 tines (Fig. 8C). Solenidia of legs I as in fig. 8D. Lengths of setae: *vi* 25 (25–30), *ve* 35 (40–45), *si* 75 (70–75), *se* 180 (170–180), *c1* 180 (175–190), *c2* 180 (180–185), *d1* 170 (150–175), *d2* 180 (150–170), *e2* 170 (150–170), *f1* 35 (25), *f2* 325 (275–300), *h1* 35 (25), *h2* (410–470), *ag1* 120 (100–115), *ag2* 70 (75–80), *ag3* 130 (135), *g1,2* 30 (20–30), *ps1,2* 20 (20), *l'RIII* 35 (40–45), *l'RIV* 35 (30–40), *3b* 40, *4b* (30), *3c* 110, *4c* 110 (90–100).

Male. Total body length 600–620 (in 5 paratypes). *Gnathosoma*. Infracapitulum apunctate. Hypostomal apex rounded, without protuberances (Fig. 8E). Movable cheliceral digits 140–145 long. Each medial branch of peritremes with 3 chambers, each lateral branch with 10 chambers (Fig. 8F). Stylophore constricted posteriorly, 180–185 long; exposed portion of stylophore apunctate, 135–140 long. *Idiosoma*. Propodonotal shield punctate laterally, weakly sclerotised, slightly concave on anterior margin, bearing bases of all propodonotal setae except *se* and *c2*. Bases of setae *se* situated anterior to level of *c1* setal bases. Length ratio of setae *vi*:*ve*:*si* 1:1.4–1.8:2.6–3.3. Hysteronotal shield fused with pygidial shield, apunctate, bearing bases of setae *d1*, *f2* and *h2*, bases of setae *e2* on or near this shield. Setae *d2* variable in length but always longer than *d1* and *e2*. All coxal fields apunctate. Length ratio of setae *ag1*:*ag2*:*ag3* 3:2:1. Setae *g1* situated anterior to bases of setae *g2* (Fig. 8H). Cuticular striations as in Figs. 8J. *Legs*. Solenidia of legs I as in Fig. 8G. Lengths of setae: *vi* 30–45, *ve* 50–65, *si* 90–115, *se* 180–210, *c1* 150–180, *c2* 195–210, *d1* 25–30, *d2* variable 80–170, *e2* 25–30, *f2* 25–30, *h2* 295–330, *ag1* 90–105, *ag2* 55–65, *ag3* 30–35, *l'RIII* 40–45, *l'RIV* 25–30, *3c* 100–105, *4c* 85–95.

### Remarks

*Charadriphilus lymnocrypte*s **n. sp.** is morphologically similar to *Ch. ludmilae* Bochkov and Chystiakov, 2001 found on the Eurasian woodcock *Scolopax rusticola* Linnaeus (Scolopacidae) from Russia (Bochkov and Chystiakov 2001). In females of both species, setae *si* are distinctly longer than *vi*; each medial branch of the peritremes has 3 or 4 chambers, the propodonotal shield is concave on the anterior margin; all coxal fields are apunctate, and fan-like setae of legs III and IV have 6–8 tines. This new species differs from *Ch. ludmilae* in the following features: in females of *Ch. lymnocryptes*, the infracapitulum is apunctate; each lateral branch of the peritremes has 9 chambers, and the lengths of setae *ve* and *si* are 35–45 and 70–75, respectively. In females of *Ch. ludmilae*, the infracapitulum is densely punctate; each lateral branch of the peritremes has 14–15 chambers, and the lengths of setae *ve* and *si* are 65 and 130, respectively.

***Niglarobia ereuneti*** **Kethley, 1970**

*Niglarobia ereuneti* Kethley, 1970: 44, figs. 25, 26. Skoracki, 2011: 290 [in key].

This species was originally described based on the mite material collected from the semipalmated sandpiper, *Calidris pusilla* (Linnaeus) (Charadriiformes: Scolopacidae) in the USA (Kethley, 1970), and so far, there have been no other records of this mite species. Below, we present a new host species and locality for this parasitic mite - the purple sandpiper *Calidris maritima* (Brünnich) from the Faroe Islands.

*Material examined*: 18 females and 5 males from quills of contour feathers from *Calidris maritima* (Charadriiformes: Scolopacidae), Nólsoy, Faroe Islands, 27 May 1996, mites collected by Simon Haarder, bird obtained by Jens-Kjeld Jensen – all specimens are deposited in the AMU, except 8 females and 3 males in the MNHD. 10 females and 2 males from quills of upper wing coverts from same host species; Nólsoy, Faroe Islands, 15 April 1996 – all specimens are deposited in the MNHD.

***Creagonycha lara*** **Kethley, 1970**

*Creagonycha lara* Kethley, 1970: 36, figs. 20, 21. Skoracki, 2011: 275 [in key].

This species was originally described based on specimens collected from the ring-billed gull, *Larus delawarensis* Ord (Charadriiformes: Laridae) in the USA (Kethley, 1970). Since its original description, no further records of this species have existed. Below, we record a new host species for this parasite, the black-legged kittiwake, *Rissa tridactyla* (Linnaeus), and a new locality, the Faroe Islands.

*Material examined*: 11 females and 1 male from quills of secondaries from *Rissa tridactyla* (Charadriiformes: Laridae), Nólsoy, Faroe Islands, 28 December 2007, mites collected by Simon Haarder, bird obtained by Jens-Kjeld Jensen. All specimens are deposited in the AMU, except 4 females in the MNHD.

**Prevalence**

A total of 31 species and 75 specimens of aquatic birds from the Faroe Islands were investigated for quill mites during this study (Table 1). Generally, only a few specimens of each bird species were examined for quill mites. Quill mites were found on five bird specimens (6%), a finding consistent with reports indicating a generally low prevalence of quill mites in avian wild populations (e.g. Skoracki et al., 2010; Skoracki, 2011). However, aquatic birds have received very little attention regarding the prevalence and intensity of quill mite infestations. Both examined specimens of the purple sandpiper (*Calidris maritima*) were infested by the quill mite *Niglarobia ereuneti* Kethley, 1970, albeit in two different microhabitats, whereas only one specimen of the northern gannet (*Morus bassanus*) was sampled for quill mites. Quill mites were recovered from one specimen of the jack snipe (*Lymnocryptes minimus*), out of three examined. Only one of the black-legged kittiwake (*Rissa tridactyla*) was found to harbour quill mites. This specimen was caught in December 2007, while the remaining kittiwakes in this study were obtained in July 2014 (1 specimen) and January 2024 (18 specimens). Further examination of additional bird specimens is imperative to attain a comprehensive understanding of the actual occurrence status of these underexplored mites on aquatic avifauna. Finally, future investigations could focus on elucidating the relationships between the presence of quill mites and seasonal fluctuations, as well as the health status, age, and sex of the host avian species.

## Data Availability

The data that support the findings of this study are available from the corresponding author upon reasonable request.
